# Aseptic meningitis in the setting of giant cell arteritis (GCA): a case report

**DOI:** 10.1186/s41927-025-00480-4

**Published:** 2025-03-10

**Authors:** Lehashenee Thirukumar, Robin Sia, Justin Jackson, John Burston

**Affiliations:** 1 Department of Medicine, Albury-Wodonga Health, Albury, NSW Australia; 2 Department of Rheumatology, Royal Melbourne Hospital, Melbourne, VIC Australia; 3 Faculty of Medicine, University of New South Wales (UNSW) Rural Clinical School, Albury, NSW Australia; 4 School of Rural Medicine, Charles Sturt University, Albury, NSW Australia

**Keywords:** Aseptic meningitis, Giant cell arteritis, Large-vessel vasculitis, Temporal arteritis, CSF

## Abstract

**Background:**

Giant cell arteritis (GCA) is a vasculitis primarily affecting medium- and large-sized arteries. The diagnosis may be challenging and lead to delays in treatment. Cerebrospinal fluid (CSF) pleocytosis is an uncommon association but may occur due to central nervous system (CNS) vasculitis or pachymeningitis. We describe a case fulfilling the criteria for diagnosing GCA, associated with CSF pleocytosis and normal neuroimaging.

**Case presentation:**

A 76-year-old woman presented to our regional hospital with three weeks of fever, confusion and fatigue. Two days later, she developed a right temporal headache with scalp tenderness. Preliminary investigations, including an FDG-PET scan, were unrevealing. Cerebrospinal fluid sampling demonstrated an isolated mononuclear pleocytosis. Brain magnetic resonance imaging (MRI) and an extensive panel of investigations failed to identify a cause, and a diagnosis of aseptic meningitis was made. An ultrasound of her right temporal artery was performed which demonstrated a non-compressible halo sign consistent with GCA. The patient was commenced on high-dose corticosteroid therapy with significant improvement in her symptoms.

**Conclusions:**

This case strengthens the association of CSF pleocytosis occurring as a complication of GCA and alerts clinicians to consider the possibility of GCA as a potential aetiology for aseptic meningitis.

**Supplementary Information:**

The online version contains supplementary material available at 10.1186/s41927-025-00480-4.

## Background

Aseptic meningitis is characterised by meningeal inflammation with cerebrospinal fluid (CSF) analysis demonstrating pleocytosis but with negative Gram staining and cultures [1]. Viruses, including enteroviruses and herpesviruses, are the most prevalent infectious cause. Non-infectious causes include medications or may be linked to systemic inflammatory disorders such as systemic lupus erythematosus (SLE), sarcoidosis, and Behçet syndrome [2].

GCA, previously known as temporal arteritis, is a form of vasculitis affecting large and medium-sized vessels. Involvement of the temporal artery and other large vessels such as branches of the aorta and vertebral arteries are the hallmark of GCA, which often manifests clinically as headache, visual abnormalities, jaw claudication, and scalp tenderness [3]. Rare complications such as tongue necrosis and tongue claudication can also occur. Constitutional symptoms, such as fever, malaise, and fatigue are not uncommon [4]. Cerebrospinal fluid pleocytosis is an uncommon association with GCA but is recognised to occur in the context of central nervous system (CNS) vasculitis or pachymeningitis. These cases have been associated with focal neurology and/or neuroimaging changes.

Here, we describe a case of aseptic meningitis without focal neurology or MRI abnormalities occurring in association with GCA.

## Case presentation

A 76-year-old Caucasian woman presented to our hospital with three weeks of quotidian low-grade fevers associated with mild chills, nausea, fatigue, loss of weight, anorexia, and confusion. Her past medical history included well-controlled type 2 diabetes, hypertension, previous cigarette smoking and a cervical spine fusion. She had no recent travel history or sick contacts. She lived at home alone and was independent in self-care.

On presentation, she was mildly confused but was not in distress. Examination of the lymphoreticular, neurological, cardiovascular, and respiratory system was unremarkable. Neck examination was limited by her previous spinal fusion. Two days after admission, she complained of a right temporal headache associated with overlying tenderness. There was no jaw claudication, visual disturbance, or proximal muscle weakness. Further examination demonstrated no carotid bruit, equal blood pressure in both arms and an absence of peripheral synovitis.

Full blood count and serum biochemistry were normal. Multiple sets of blood cultures were negative. Her C-reactive protein (CRP) was 148 mg/L and her erythrocyte sedimentation rate (ESR) was 58 mm/hr. An extensive panel of investigations was conducted as described in Table 1.

Computer tomography (CT) of the chest, abdomen, and pelvis as well as fluorodeoxyglucose-positron emission tomography (FDG-PET) did not identify an occult source of infection or malignancy. Mildly avid bilateral mediastinal and hilar lymph nodes were seen on the FDG-PET, raising the possibility of neurosarcoidosis. The patient proceeded to bronchoscopy with endobronchial biopsy which yielded only benign epithelium and collagenous tissue and no evidence of granulomatous inflammation.

**Fig. 1:**
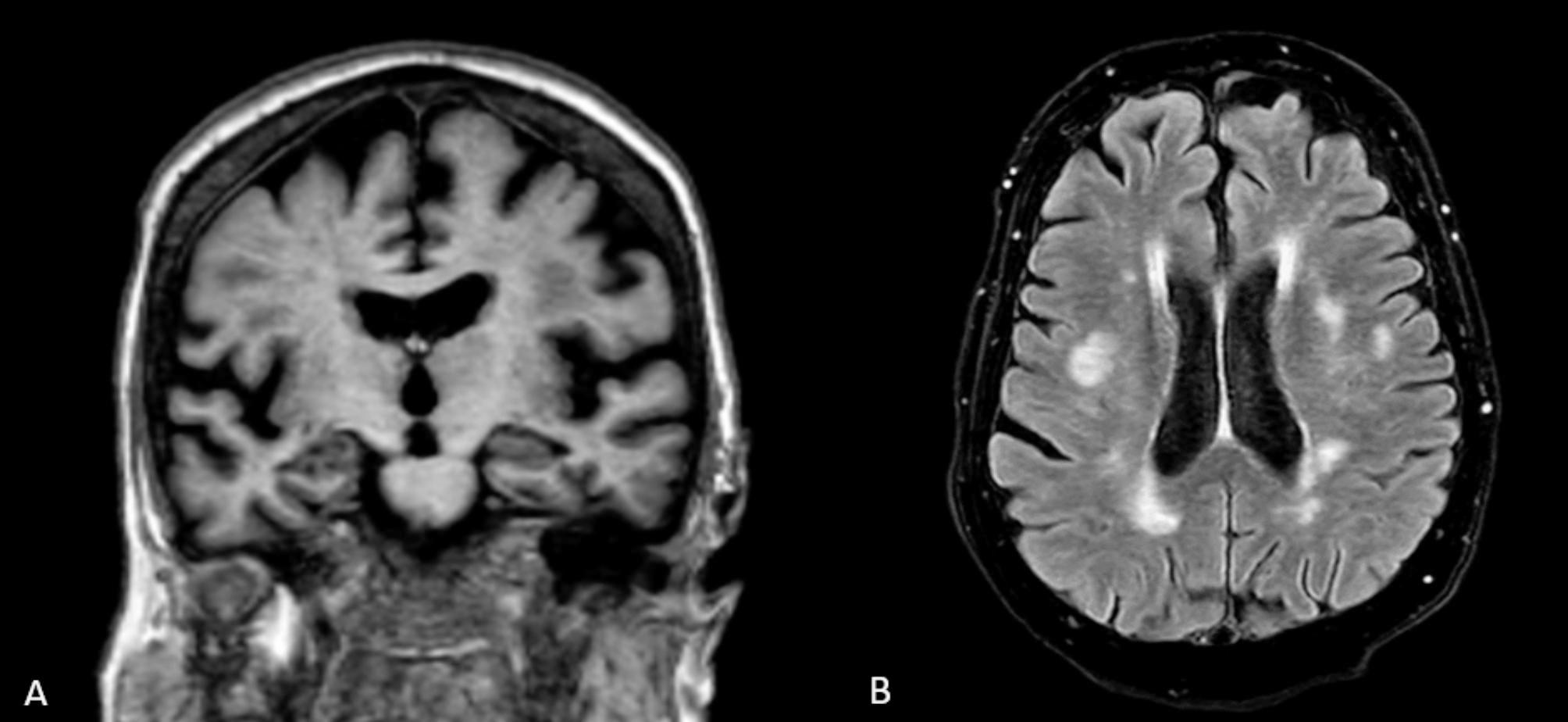
Non-contrast MRI of the brain on day 65 of admission. (**A**) Coronal view with age-related parenchymal changes without focal hippocampal atrophy. (**B**) Axial view showing mild chronic small vessel ischemic changes in the cerebral white matter

CSF analysis revealed a normal protein and glucose with a lymphocytic pleocytosis of 48 × 10^6^ cells per litre. The Gram stain was negative, there was no growth on CSF cultures and a viral PCR panel was also negative. A diagnosis of aseptic meningitis was made. Magnetic resonance imaging (MRI) of the brain performed on day 2 (without contrast) and on day 65 (with and without contrast) excluded pachymeningitis, identifying only stable chronic small vessel ischemic changes (Fig. 1).

**Table 1:** Diagnostic investigations undertaken which were negative or within normal limits

Autoimmune markers	Paraneoplastic and neuronal antibodies	Infective Screening	Other serum markers	CSF analysis	Broncho-alveolar lavage
Anti-CCP Rheumatoid factor Anti-dsDNA ANA MPO-ANCA PR3-ANCA ENA antibodies HLA-B*51	ANNA-1/anti-Hu ANNA-2/anti-Ri Anti-PCA-1/Yo Anti-PCA-2 Anti-CV2/CRMP5 Anti-PCA-Tr Anti-Ma/Ta Anti-GAD Anti-Amphiphysin	HIV serology Syphilis serology Respiratory viral PCR CMV PCR EBV PCR TB IGRA Whipple’s serology	Serum ACE LDH Creatinine kinase Serum PEP and free light chains TSH Vitamin B12	Enterovirus, HSV, VZV, EBV and CMV PCR Cryptococcal antigen Fungal culture Flow cytometry	Bacterial culture AFB staining and culture Atypical pneumonia PCR Cytology Histopathology Flow cytometry

Note: Serum ammonia and immunoglobulin (Ig)G4 levels were not measured

Abbreviations: CCP, Cyclic citrinullated peptide; dsDNA, Double stranded DNA; ANA, Antinuclear antibody; MPO-ANCA, Myeloperoxidase-antineutrophil cytoplasm autoantibody; PR3-ANCA, Proteinase 3-antineutrophil cytoplasm autoantibody; ENA, Extractable nuclear antigen; PCA, Purkinje cell cytoplasmic antibody; CRMP5, Collapsin response mediator protein 5; GAD, Glutamic acid decarboxylase; IGRA, Interferon-gamma release assay; ACE, Angiotensin converting enzyme; LDH, Lactate dehydrogenase; PEP, Protein electrophoresis; TSH, Thyroid-stimulating hormone; HSV, Herpes simplex; VZV, Varicella-Zoster; EBV, Epstein–Barr virus; CMV, Cytomegalovirus; PCR, Polymerase chain reaction; AFB, Acid fast bacilli

**Fig. 2:**
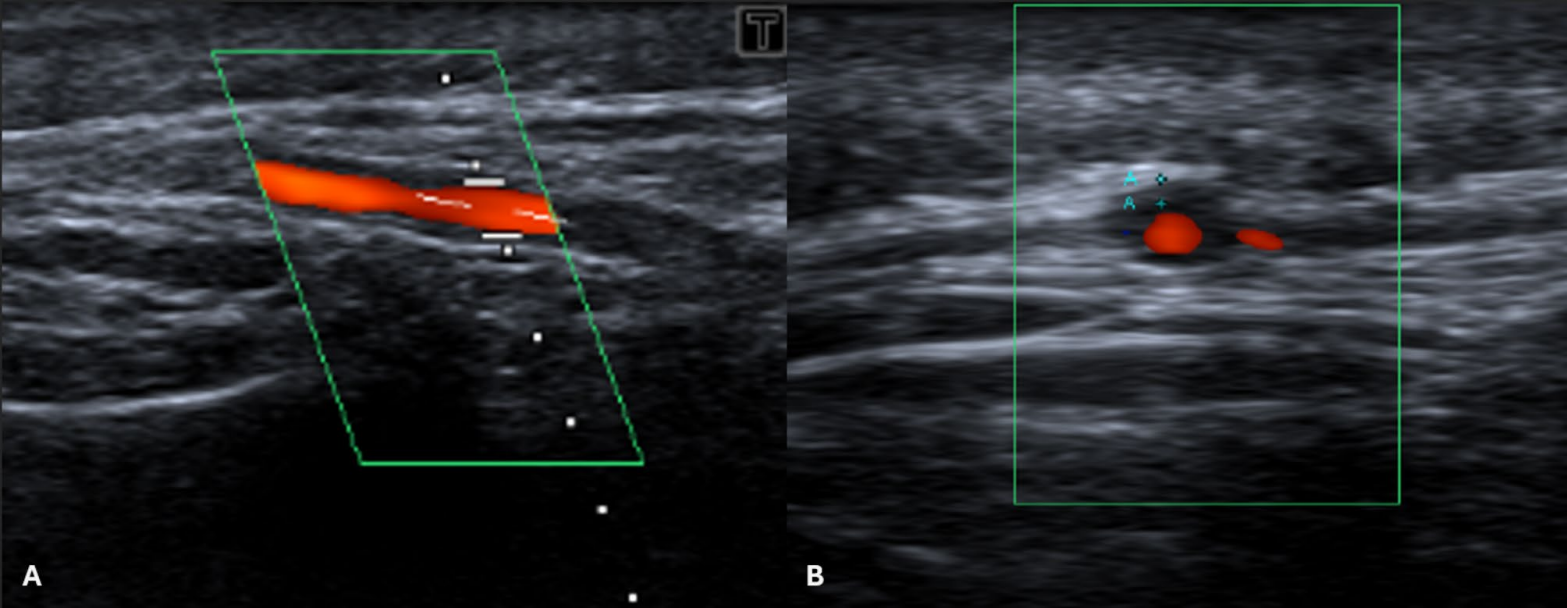
Duplex ultrasound of the right temporal artery. (**A**) Longitudinal view with visible thickening of the vessel wall. (**B**) Transverse view with halo sign indicative of GCA

In view of the development and persistence of the right temporal headache, an ultrasound of the right temporal artery was performed. It demonstrated a non-compressible hypoechoic halo sign of 0.5 mm – exceeding the normal cut-off of 0.42 mm [5] – suggestive of GCA (Fig. 2).

The patient was commenced on intravenous methylprednisolone 1 g for three days and subsequently treated with oral prednisolone 60 mg daily with an ongoing weaning dose. She demonstrated significant improvement in her right temporal headache within days. Her fever, which had resolved within the first week of admission, remained absent. Inflammatory markers, including CRP and ESR, showed a downward trend and eventually normalised. A repeat lumbar puncture was not performed, and therefore, CSF was not retested post-treatment. However, despite these improvements, the patient’s confusion persisted. She developed persistent cognitive impairment, which required ongoing supportive management.

## Discussion and conclusions

Aseptic meningitis, a syndrome with an acute or subacute onset, is defined as meningeal inflammation consisting of clinical features of meningitis, such as fever, headache, nausea or vomiting and neck stiffness, in conjunction with a CSF pleocytosis and negative Gram stain and cultures [1]. Our patient fulfilled the clinical and CSF criteria for aseptic meningitis.

The 2022 ACR/EULAR Classification Criteria state that a diagnosis of GCA can only be made in patients aged 50 and above [3]. According to these classification criteria, either a positive temporal artery biopsy or a halo sign elicited on temporal artery ultrasound generates the greatest diagnostic weight, scoring 5 points. Additional points are given for evidence of extracranial vessel involvement, such as visual loss, jaw claudication or scalp tenderness, as well as investigation findings including a raised ESR or CRP [3]. Our patient’s presentation scored 12 points, well above the 6 points required to support the diagnosis of GCA. In patients with a high pre-test probability and positive imaging findings, additional investigations such as temporal artery biopsy may not be required [6].

Given the challenging assessment of a confused patient, rare association of CSF pleocytosis with GCA and an FDG-PET scan which did not reveal evidence of vasculitis, there was a delay in confirming the diagnosis in our patient. While the 2023 EULAR recommendations state that FDG-PET can be used as an alternative for evaluating cranial arteries in patients with possible GCA, ultrasound of the temporal and axillary arteries remains the first-line imaging modality [6]. Ultrasound has been shown to be more sensitive at detecting GCA than FDG-PET. In low risk-of-bias (RoB) studies, ultrasound had a greater pooled sensitivity than FDG-PET at 88% compared to 76% [7]. This is supported by Nielsen et al., the only low RoB study that directly compared ultrasound with FDG-PET, and reported a sensitivity of 91% for ultrasound compared to 79% for FDG-PET when assessing both cranial and extracranial arteries [8].

Neurologic complications in GCA are uncommon, and typically manifest as neuropathies or, rarely, ischemic strokes [9–15]. CSF abnormalities have previously been reported in GCA, including hyperproteinorrachia, elevated CSF interleukin-6 levels and mononuclear pleocytosis [11–20]. In contrast to our patient, CSF pleocytosis is often identified in association with focal neurology and evidence of intracranial vasculitis and ischaemic changes visualised on MRI [11–15]. A case report from 1980, prior to the widespread availability of MRI, reports an abnormal radioisotope brain scan and CSF findings consistent with intracranial vasculitis in association with GCA [16]. Patients have also been described with GCA-associated hypertrophic pachymeningitis, where, in addition to CSF abnormalities, contrast-enhanced MRI revealed enhancing dural thickening [19, 20]. Kutty et al. postulate that vascular inflammation of the dural arteries may result in dural thickening and CSF pleocytosis [19, 20]. Roelcke et al. reported a case of meningoradiculitis resulting in bilateral cranial nerve VII palsies with a CSF pleocytosis in association with giant cell arteritis [18].

Our case provides evidence that temporal arteritis may also result in a CSF pleocytosis in the absence of focal neurology or neuroimaging changes. Aseptic meningitis may result in leptomeningeal contrast enhancement on MRI, but is often absent in cases with little to no dysfunction of the blood-CSF barrier, which was likely in our patient given her normal CSF protein levels [21].

Table 2 describes the reported cases of CSF pleocytosis in association with GCA and illustrates the spectrum of neurological and clinical manifestations.

**Table 2:** Cases of GCA with CSF pleocytosis and their associated neurological findings

Authors	Patient age	GCA features	Focal neurology	CSF findings	Neuroimaging
Hirsch et al [11]	47	Headache, fever, weight loss, fatigue, elevated ESR	Left hemiparesis	Hyperproteinorrachia and pleocytosis	Angiography: marked stenoses in intracranial arteries
Salvarani et al [12]	72 (case 2)	Headache, jaw claudication, elevated ESR	Spastic dysarthria, apraxia of speech, gait ataxia	Hyperproteinorrachia and pleocytosis	MRI: multiple widespread ischaemic infarcts
Larivière et al [13]	59 (case 2)	Headache, fever, weight loss, jaw claudication, scalp dysesthesia, diplopia, amaurosis, elevated CRP	Left hemidysesthesia, and horizontal diplopia	Pleocytosis	MRI: multiple small acute ischemic lesions, left frontal lobe ischaemia
	72 (case 4)	Headache, weight loss, jaw claudication, scalp hyperesthesia, left amaurosis, elevated CRP	Left upper limb paresis, gait ataxia	Hyperproteinorrachia and pleocytosis	MRI: bilateral cerebellar infarcts
Parra et al [14]	56	Headache, weight loss, tender temporal arteries, raised CRP and ESR	Left central facial palsy, spastic tetraparesis, impaired awareness	Pleocytosis	MRI: multiple watershed infarcts, intracranial dissections
Roelcke et al [18]	63	Headache, neck stiffness, fever, jaw claudication, tender temporal artery, raised ESR	Bilateral 7th nerve palsy	Hyperproteinorrachia and pleocytosis	MRI: right cerebellar tentorium meningeal enhancement; normal parenchyma
Kutty et al [20]	53	Headache, fever, right orbital pain, raised CRP and ESR	Absent	Hyperproteinorrachia and pleocytosis	MRI: focal enhancement of dura
Present case	76	Headache, fever, weight loss, fatigue, scalp tenderness, elevated CRP and ESR	Absent	Pleocytosis	MRI: no acute infarct

Although GCA-induced cognitive impairment may be reversed when treatment is initiated early, our patient’s lingering confusion and cognitive deficits may have been caused by irreversible neural injury that occurred during the diagnostic delay. In GCA, the intense inflammatory process – exacerbated by age-related vascular changes – can cause microvascular damage and neuronal injury resulting in neurological aging that may not recover even after systemic inflammation is controlled [22]. Some reports, such as by Lahaye et al., demonstrated significant cognitive recovery with early intervention [23]. However, delayed treatment or pre-existing cerebrovascular pathology may lead to permanent deficits [22–24]. Additionally, while strokes are well-known causes of cognitive decline in GCA [24], our patient’s MRIs did not reveal any new infarcts, suggesting that a more subtle, diffuse inflammatory insult may be responsible. This is similar to the neuropsychiatric sequelae observed in a small case series of aseptic meningitis, where some patients experienced persistent cognitive impairment despite the resolution of acute inflammation [25]. Thus, even though the clinical and biochemical features of GCA improved, the irreversible damage incurred during the acute phase may account for her enduring cognitive impairment. Furthermore, the age-related involutional changes observed on the initial MRI Brain may have represented heretofore subclinical cognitive decline which was exacerbated by the protracted delirium associated with her illness.

Despite our extensive investigations, we acknowledge the possibility of an explanation other than GCA for the CSF pleocytosis present in our patient. However, the temporal association, lack of an alternate diagnosis and other reports of CSF pleocytosis in association with GCA strengthen the probability that GCA was the causal pathology.

In conclusion, our case alerts clinicians to the importance of considering GCA as a potential cause of aseptic meningitis and strengthens the associations between GCA and a CSF pleocytosis. Ultrasound of the temporal and axillary arteries represents a non-invasive and relatively inexpensive investigation modality and was key to achieving a diagnosis in our patient. Timely recognition and prompt initiation of steroids is necessary in GCA to prevent clinical deterioration and complications.

## Electronic supplementary material

Below is the link to the electronic supplementary material.

Supplementary Material 1

Supplementary Material 2

Supplementary Material 3

Supplementary Material 4

## Data Availability

The datasets generated or analysed during the present study are included within the article and the remaining are available from the corresponding authors on reasonable request.

## Abbreviations

ACE: Angiotensin converting enzyme
ACR/EULAR: American college of rheumatology/European league against rheumatism
ANA: Antinuclear antibody
CCP: Cyclic citrinullated peptide
CRP: C-reactive protein
CNS: Central nervous system
CSF: Cerebrospinal fluid
CT: Computed tomography
dsDNA: Anti-double stranded DNA
ENA: Extractable nuclear antigen
HLA: Human leukocyte antigen
ESR: Erythrocyte sedimentation rate
FDG-PET: Fluorodeoxyglucose-positron emission tomography
GCA: Giant cell arteritis
LDH: Lactate dehydrogenase
SPEP: Serum protein electrophoresis
MPO-ANCA: Myeloperoxidase-antineutrophil cytoplasm autoantibody
PR3: Proteinase 3
MRI: Magnetic resonance imaging
PCR: Polymerase chain reaction
RoB: Risk-of-bias
SLE: Systemic lupus erythematosus

## References

[1] Tapiainen T, Prevots R, Izurieta HS, Abramson J, Bilynsky R, Bonhoeffer J, et al. Aseptic meningitis: case definition and guidelines for collection, analysis and presentation of immunization safety data. Vaccine. 2007;25(31):5793–802. 17574313 10.1016/j.vaccine.2007.04.058

[2] Shukla B, Aguilera EA, Salazar L, Wootton SH, Kaewpoowat Q, Hasbun R. Aseptic meningitis in adults and children: diagnostic and management challenges. J Clin Virol. 2017;94:110–4. 28806629 10.1016/j.jcv.2017.07.016 PMC5581214

[3] Ponte C, Grayson PC, Robson JC, Suppiah R, Gribbons KB, Judge A, et al. 2022 American college of rheumatology/eular classification criteria for giant cell arteritis. Ann Rheum Dis. 2022;81(12):1647. 36351706 10.1136/ard-2022-223480

[4] Dejaco C, Duftner C, Buttgereit F, Matteson EL, Dasgupta B. The spectrum of giant cell arteritis and polymyalgia rheumatica: revisiting the concept of the disease. Rheumatology. 2017;56(4):506–15. 27481272 10.1093/rheumatology/kew273

[5] Schäfer VS, Juche A, Ramiro S, Krause A, Schmidt WA. Ultrasound cut-off values for intima-media thickness of Temporal, facial and axillary arteries in giant cell arteritis. Rheumatology. 2017;56(9):1479–83. 28431106 10.1093/rheumatology/kex143

[6] Dejaco C, Ramiro S, Bond M, Bosch P, Ponte C, Mackie SL, et al. EULAR recommendations for the use of imaging in large vessel vasculitis in clinical practice: 2023 update. Ann Rheum Dis. 2024;83(6):741. 37550004 10.1136/ard-2023-224543

[7] Bosch P, Bond M, Dejaco C, Ponte C, Mackie SL, Falzon L, et al. Imaging in diagnosis, monitoring and outcome prediction of large vessel vasculitis: a systematic literature review and meta-analysis informing the 2023 update of the EULAR recommendations. RMD Open. 2023;9(3):e003379. 37620113 10.1136/rmdopen-2023-003379 PMC10450079

[8] Nielsen BD, Hansen IT, Keller KK, Therkildsen P, Gormsen LC, Hauge E-M. Diagnostic accuracy of ultrasound for detecting large-vessel giant cell arteritis using FDG PET/CT as the reference. Rheumatology. 2019;59(8):2062–73. 10.1093/rheumatology/kez568 31808526

[9] Rahman W, Rahman FZ. Giant cell (Temporal) arteritis: an overview and update. Surv Ophthalmol. 2005;50(5):415–28. 16139037 10.1016/j.survophthal.2005.06.011

[10] Smith JH, Swanson JW. Giant cell arteritis. Headache. 2014;54(8):1273–89. 25041449 10.1111/head.12425

[11] Hirsch M, Mayersdorf A, Lehmann E. Cranial giant-cell arteritis. Br J Radiol. 1974;47(560):503–6. 4547494 10.1259/0007-1285-47-560-503

[12] Salvarani C, Giannini C, Miller DV, Hunder G. Giant cell arteritis: involvement of intracranial arteries. Arthritis Rheum. 2006;55(6):985–9. 17139651 10.1002/art.22359

[13] Larivière D, Sacre K, Klein I, Hyafil F, Choudat L, Chauveheid M-P, et al. Extra- and intracranial cerebral vasculitis in giant cell arteritis: an observational study. Medicine. 2014;93(28). 10.1097/MD.0000000000000265 PMC4603113 25526454

[14] Parra J, Domingues J, Sargento-Freitas J, Santana I. Extensive intracranial involvement with multiple dissections in a case of giant cell arteritis. BMJ Case Rep. 2014;2014. 10.1136/bcr-2014-204130 PMC3987556 24728901

[15] Beuker C, Wankner MC, Thomas C, Strecker J-K, Schmidt-Pogoda A, Schwindt W, et al. Characterization of extracranial giant cell arteritis with intracranial involvement and its rapidly progressive subtype. Ann Neurol. 2021;90(1):118–29. 33993547 10.1002/ana.26101

[16] Gordon M, Gordon EP. Temporal arteritis: cerebrospinal fluid and brain scan abnormalities (Case Report). J Am Geriatr Soc. 1980;28(3):136–8. 7354208 10.1111/j.1532-5415.1980.tb00248.x

[17] Hirohata S, Tanimoto K, Ito K. Elevation of cerebrospinal fluid Interleukin-6 activity in patients with vasculitides and central nervous system involvement. Clin Immunol Immunopathol. 1993;66(3):225–9. 8432047 10.1006/clin.1993.1029

[18] Roelcke U, Eschle D, Kappos L, Moschopulos M, Laeng RH, Buettner UW. Meningoradiculitis associated with giant cell arteritis. Neurology. 2002;59(11):1811–2. 12473783 10.1212/01.wnl.0000039924.73270.89

[19] Boisch G, Duda S, Hartmann C, Weßling H. Hypertrophic pachymeningoencephalitis associated with temporal giant cell arteritis. BMJ Case Rep. 2018;2018. 10.1136/bcr-2018-225304 PMC6169653 30262524

[20] Kutty RK, Maekawa M, Kawase T, Fujii N, Kato Y. Temporal arteritis with focal pachymeningitis: a deceptive association. Nagoya J Med Sci. 2020;82(1):143–50. 32273643 10.18999/nagjms.82.1.143 PMC7103863

[21] Alonso A, Eisele P, Ebert AD, Griebe M, Engelhardt B, Szabo K, et al. Leptomeningeal contrast enhancement and blood-CSF barrier dysfunction in aseptic meningitis. Neurol Neuroimmunol Neuroinflammation. 2015;2(6):e164. 10.1212/NXI.0000000000000164 PMC4608759 26516629

[22] Watanabe R, Hashimoto M. Aging-Related vascular inflammation: giant cell arteritis and neurological disorders. Front Aging Neurosci. 2022;14. 10.3389/fnagi.2022.843305 PMC9039280 35493934

[23] Lahaye C, Sanchez M, Rouet A, Gross A, Faucher N, Raynaud-Simon A, et al. A curable pseudo-dementia related to an atypical presentation of giant cell arteritis. Age Ageing. 2020;49(3):487–9. 32147681 10.1093/ageing/afaa010

[24] Solans-Laqué R, Bosch-Gil JA, Molina-Catenario CA, Ortega-Aznar A, Alvarez-Sabin J, Vilardell-Tarres M. Stroke and Multi-Infarct dementia as presenting symptoms of giant cell arteritis: report of 7 cases and review of the literature. Medicine. 2008;87(6). 10.1097/MD.0b013e3181908e96 19011505

[25] Gunst J, Andersen H, Hjerrild S, Marinovskij E, Deutch S, Schembri A, et al. Neuropsychiatric symptoms among adult patients with aseptic meningitis: A prospective case series. Acta Psychiatrica Scandinavica. 2015;133. 10.1111/acps.12539 26685715

