# Supplementary figures and images for: Aseptic meningitis in the setting of giant cell arteritis (GCA): a case report
Source: BMC Rheumatol. 2025 Mar 10;9:29. doi: 10.1186/s41927-025-00480-4 (PMC11892150; doi:10.1186/s41927-025-00480-4)

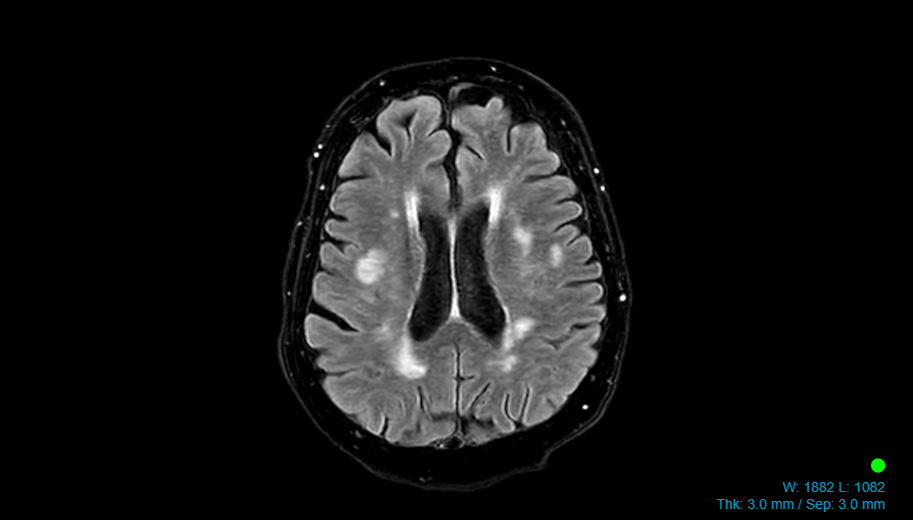

Supplement: Supplementary file 1 — Supplementary Material 1 [file 41927_2025_480_MOESM1_ESM.png]

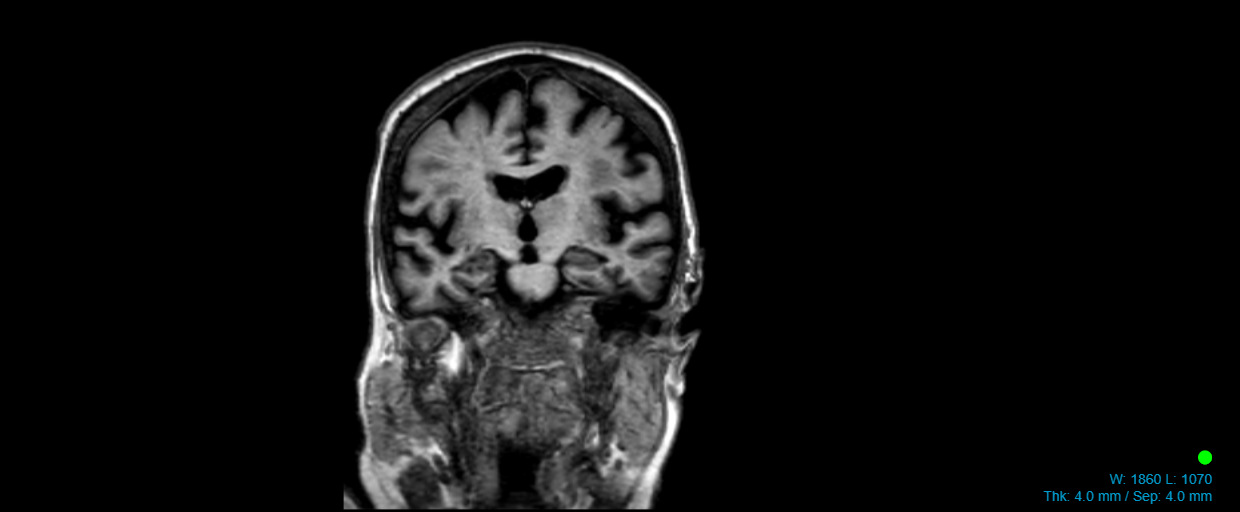

Supplement: Supplementary file 2 — Supplementary Material 2 [file 41927_2025_480_MOESM2_ESM.png]

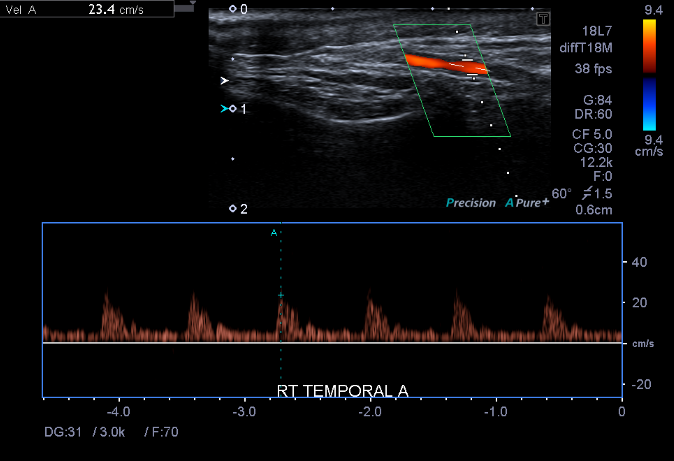

Supplement: Supplementary file 3 — Supplementary Material 3 [file 41927_2025_480_MOESM3_ESM.png]

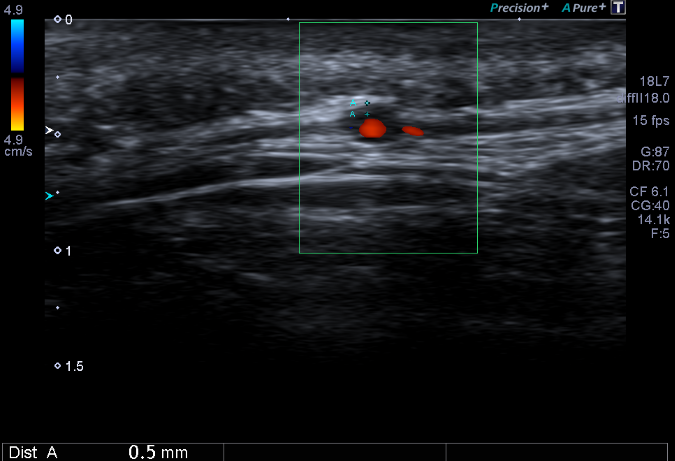

Supplement: Supplementary file 4 — Supplementary Material 4 [file 41927_2025_480_MOESM4_ESM.png]
